# Sleep Disturbances in Chronic Kidney Disease – The CKD-REIN Cohort Study

**DOI:** 10.1016/j.xkme.2026.101530

**Published:** 2026-09-15

**Authors:** Bénédicte Stengel, Emilie Moutard, Marie Metzger, Isabelle Jaussent, Aghilès Hamroun, Solène M. Laville, Abdou Omorou, Denis Fouque, Ziad A. Massy, Roberto Pecoits-Filho, Luc Frimat, Natalia Alencar de Pinho, Sophie Liabeuf, CKD-REIN study investigators

**Affiliations:** 1 Centre for Research in Epidemiology and Population Health (CESP), Paris-Saclay University, Inserm U1018, Versailles Saint-Quentin University, Clinical Epidemiology Team, Villejuif, France; 2 University of Montpellier, Institute for Neurosciences of Montpellier (INM), Inserm, Montpellier, France; 3 Service de Santé Publique, Epidémiologie – Maison Régionale de la Recherche Clinique, CHU Lille, Lille, France; 4 UMR1167 RIDAGE, Institut Pasteur de Lille, Inserm, Univ Lille, Lille, France; 5 Pharmacoepidemiology Unit, Department of Clinical Pharmacology, Amiens-Picardie University Medical Center, Amiens, France; 6 MP3CV Laboratory, Jules Verne University of Picardie, Amiens, France; 7 CHRU-Nancy, INSERM, Université de Lorraine, CIC-1433 Epidémiologie Clinique, Nancy, France; 8 UMR 1319 INSPIIRE, Inserm, Université de Lorraine, Nancy, France; 9 Department of Nephrology, CHU Lyon-Sud, Université de Lyon, Lyon, France; 10 Inserm U1060, CARMEN, Lyon, France; 11 Association pour l'Utilisation du Rein Artificiel (AURA) Paris, and Nephrology Department, Ambroise Paré University Hospital, APHP, Boulogne-Billancourt, Paris, France; 12 ClinEpi Program Area, Arbor Research Collaborative for Health, Ann Arbor, MI USA; 13 Service de Néphrologie, CHRU de Nancy, F-54000 Vandoeuvre-lès-Nancy, France

**Keywords:** Cramps, drug-induced effects, excessive daytime sleepiness, fatigue, glomerular filtration rate, insomnia, itching

## Abstract

**Rationale & Objective:**

Insomnia and excessive daytime sleepiness (EDS) are common in patients with chronic kidney disease (CKD), but their determinants are poorly understood. We analyzed multiple factors likely to affect sleep with the aim of disentangling the contributions of CKD markers and symptoms from those of social factors, lifestyle, comorbid conditions, and drug-induced effects, with the latter 2 highly prevalent in this population.

**Study Design:**

Prospective cohort study

**Setting & Participants:**

In total, 2,765 patients with CKD stages 2-5 not receiving dialysis (mean age, 67 ± 13 years; 66% men) from a representative sample of 40 nephrology clinics in France.

**Exposures:**

Albuminuria, estimated glomerular filtration rate, CKD symptoms, lifestyle, comorbid conditions, and medications collected annually.

**Outcomes:**

Insomnia and EDS ≥ 3 times/week in the past 3 months assessed with a standardized questionnaire at baseline, 1-, 3-, and 5-year follow-ups*,* providing 7,264 observations overall.

**Analytical Approach:**

Adjusted odds ratios of insomnia and EDS associated with time-updated exposures estimated with mixed effects logistic regressions.

**Results:**

Baseline prevalence of reported insomnia was 22% (95% confidence interval, 21%-24%) and that of EDS, 15% (14%-16%). Based on the 7,264 observations, neither prevalence was associated with albuminuria after adjustment for age and gender. The odds ratio of EDS alone increased significantly with decreasing estimated glomerular filtration rate, 1.16 (1.05-1.28 mL/min/1.73 m^2^) per 10 mL/min/1.73 m^2^, independent of social factors, physical activity, obesity, heart failure, and long half-life benzodiazepine use. It was no longer significant after adjustment for fatigue, which displayed a strong dose–effect relation with EDS, depression, insomnia, and sleep apnea. Insomnia prevalence increased strongly with severity of cramps, itching, and stress, independent of other determinants including female gender, heart failure, thyroid hormone use, depression, and sleep apnea.

**Limitations:**

Self-reported sleep disturbances and limited longitudinal inference.

**Conclusions:**

This study highlights the multifactorial nature of sleep disturbances in CKD and the potentially modifiable contribution of CKD symptoms and drug-induced effects.

## Plain-Language Summary

Sleep disturbances are common in patients with chronic kidney disease (CKD), but the causes are poorly understood. We studied multiple factors likely to affect sleep so that we could disentangle the specific contribution of CKD from those of social factors, lifestyle, comorbid conditions, and drug-induced effects in the prevalence of insomnia and excessive daytime sleepiness. We found that patients with CKD-related cramps, itching, and stress were more likely to report insomnia, regardless of other factors, whereas those with CKD-related fatigue or using long half-life benzodiazepine often reported excessive daytime sleepiness. This study highlights not only the multifactorial nature of sleep disturbances in CKD but also the potentially modifiable contribution of disease symptoms and inappropriate drug prescriptions, which must be considered in this population.

Sleep is essential for health through its involvement in metabolism, immune and hormone functions, and the cardiovascular system.^1^ Population-based studies provide consistent findings linking short sleep duration and sleep disturbances, including insomnia and excessive daytime sleepiness (EDS), with increased risk of cardiometabolic and neurodegenerative diseases, as well as with impaired quality of life, mental health, and daily functioning.^1^, ^2^, ^3^, ^4^, ^5^, ^6^ Evidence suggests that sleep disturbances are common in chronic kidney disease (CKD)^7^, ^8^, ^9^, ^10^ and associated with adverse kidney and cardiovascular outcomes.^11^, ^12^, ^13^, ^14^ Although insomnia and EDS are common and well-documented in patients receiving dialysis, their prevalence and determinants are poorly understood at earlier stages of CKD.^8^^,^^14^^,^^15^ Prevalence estimates vary widely depending on the population studied, the definitions, and methods used to assess sleep disorders.^4^^,^^9^^,^^16^, ^17^, ^18^ Meta-analyses in individuals with CKD not receiving dialysis show pooled prevalence rates of 48% (95% confidence interval [CI], 30%-67%) for insomnia symptoms (4 studies) and 22% (CI, 18%-28%) for EDS (7 studies).^8^^,^^9^

Several factors have been associated with either or both insomnia and EDS, including age, gender, social factors, lifestyle, smoking, physical activity, medications affecting sleep, obesity, diabetes, anxiety, depression, and obstructive sleep apnea.^4^^,^^14^^,^^15^^,^^17^, ^18^, ^19^ Whether these factors, common in patients with CKD, primarily explain their high prevalence of sleep disturbances or if CKD-related factors also contribute remains unknown. One explanation for this lack of knowledge is that studies examining the relation of insomnia and EDS with estimated glomerular filtration rate (eGFR) and albumin-to-creatinine ratio (ACR) levels are scarce and that their findings mixed.^9^^,^^10^^,^^20^^,^^21^ From this perspective, it is important to emphasize that the concept of a bidirectional relation between CKD and sleep disorders^14^^,^^22^ is currently supported by studies showing the impact of sleep on CKD incidence or progression^11^, ^12^, ^13^, ^14^^,^^23^; however, that evidence for the existence of a reverse association, ie, a potential effect of CKD on sleep, mainly relies on studies in patients receiving dialysis.^8^^,^^14^^,^^22^ A second reason is that no studies have examined the relation of sleep disturbances with the most frequent and bothersome CKD symptoms, such as fatigue, cramps, and itching,^7^^,^^24^ or with cardiovascular complications^25^ and cognitive impairment, which are also common in CKD.^26^^,^^27^

We used the CKD-Renal Epidemiology and Information Network (CKD-REIN) cohort study^28^ of patients with moderate to severe CKD to analyze multiple factors likely to affect sleep with the aim of disentangling the contributions of CKD markers and symptoms from those of social factors, lifestyle, comorbid conditions, and drug-induced effects in the prevalence of insomnia and EDS.

## Methods

### Study Design and Population

The CKD-REIN cohort included 3033 adult patients with all-cause CKD stages 2-5, neither receiving dialysis nor transplanted. They were enrolled in 40 randomly selected nephrology outpatient clinics in France and followed up from 2013 to 2020. A detailed description of the study design has been published and is registered on ClinicalTrials.gov (Identifier: NCT03381950).^29^ The French National Institute of Health and Medical Research institutional review board (IRB00003888) and the Ethics Committee (CCTIRS12.360/CPP) approved the study. All patients provided written informed consent.

### Data Collection

We collected data annually from patient records, interviews by trained clinical research associates, and self-administered questionnaires for 5 years. These included sociodemographic factors, living condition (alone or not), occupational status (employed, retired, unemployed), smoking, physical activity (assessed using the Global Physical Activity Questionnaire),^30^ hypertension (reported in medical records or treated), diabetes (reported in medical record or treated, HbA1c ≥6.5%, fasting glucose ≥7.0 mmol/L, or a random glucose ≥11.0 mmol/L), heart failure, other cardiovascular diseases (atheromatous cardiovascular disease and arrhythmias), and chronic respiratory disease. A diagnosis of sleep apnea with or without use of continuous positive airway pressure was also recorded from medical records at baseline. At each visit, patients were asked to provide all their drug prescriptions, from 3 months before inclusion until the end of follow-up. Drug classes were coded by the international Anatomical Therapeutic and Chemical thesaurus.^31^ A pharmacologist (SL) established a comprehensive list of all medications known for their potential effect on sleep (Table S1). Body mass index (kg/m^2^), blood pressure (BP, mm Hg), serum creatinine, and albuminuria were measured at each visit; routine laboratory measurements were also recorded. GFR was estimated with the 2009 creatinine-based Chronic Kidney Disease Epidemiology Collaboration (CKD-EPI) equation.^32^ Albuminuria was either measured or estimated by equation from proteinuria.^33^ Cognitive function was assessed at baseline with the Mini-Mental State Examination (MMSE) form, and cognitive impairment defined by a MMSE score <24.^34^

We used the Kidney Disease Quality of Life Questionnaire to assess symptoms, including cramps, itchy skin, fatigue (feeling washed out or drained), and stress or worries about kidney disease, using the following question: “During the past 4 weeks, to what extent were you bothered by (this symptom): not at all, somewhat, moderately, very much, or extremely?” The Center for Epidemiological Studies-Depression (CES-D) scale^35^^,^^36^ was used to assess depression, defined as a score ≥10 or antidepressant drug prescriptions. Response rates to the patient questionnaires ranged from 89% at baseline to 67% at the 5-year follow-up.

### Assessment of Sleep Duration and Disturbances

To assess sleep quality, patients were requested to complete standardized questions from the Basic Nordic Sleep Questionnaire at baseline and the 1-, 3-, and 5-year follow-ups; 2,765 patients (91%) completed at least one questionnaire and provided 7,264 responses overall.^37^ The questions focused on sleep duration and concerns in the past 3 months and were all scored on a scale ranging from 1 (never or less than once a month) to 5 (almost daily). Reported mean duration of nighttime sleep (in hours and minutes) and the frequency of naps with their duration were used to estimate nighttime, daytime, and 24-hour sleep durations.

Insomnia was defined as “having slept poorly or rather poorly over the last 3 months, together with having had difficulty falling asleep, waking up in the night or waking up too early in the morning without being able to go back to sleep at least 3 days per week.” EDS was defined as “feeling very sleepy during the day at least 3 days per week.” Patient-reported sleep apnea was recorded concurrently and defined as “having breathing pauses during sleep (sleep apnea) noticed by others at least 3 days per week.” Finally, patients were asked about their frequency of “use of sleeping pills prescribed by a doctor.”

### Statistical Analyses

Baseline mean 24-hour, nighttime, and median daytime sleep durations, as well as the prevalence of sleep disturbances, were compared between men and women, age groups (<65, 65-75, and ≥75 years), and categories of eGFR (≥45, 30-44, and <30 mL/min/1.73 m^2^), ACR (<30, 30-300, and ≥300 mg/g), and CKD risk (low or moderately increased, high, and very high), with the χ^2^ test and the Cochran–Armitage test for trend.

All 7,264 observations were used in mixed effect logistic regressions with random intercepts to estimate the odds ratios (ORs) and 95% CIs of sleep disturbances associated with the factors studied, with correlations between repeated measurements taken into account. All clinical, laboratory, and treatment data were updated at the time of completion of the annual patient questionnaire, except the MMSE score, available at baseline only. To address the study aims, we conducted step-by-step analyses, first by testing the effect of time (in years), modeled as a continuous fixed effect, on the occurrence of insomnia and EDS. Second, we estimated ORs adjusted for age, gender, eGFR (per 10 mL/min/1.73 m^2^), and ACR (per 2-fold increase; the 4-variable-adjusted model) associated with each risk factor identified from literature review,^4^^,^^14^, ^15^, ^16^, ^17^^,^^19^ each relevant CKD symptom, and medication listed for its potential effect on sleep (models for insomnia also included time). Third, we performed a multivariable model including these 4 variables (age, gender, eGFR, and ACR, and time for insomnia) as well as social and lifestyle factors, comorbid conditions, and medications specific for each sleep disorder when their *P* value was <0.10 in the 4-variable adjusted model. Finally, in a last full model, we included the 4 variables and time as mentioned above, CKD symptoms, factors with a *P* value < 0.10 in the previous model, depression, sleep apnea for insomnia, and both insomnia and sleep apnea for EDS. Interactions with gender were tested for all associations studied.

To manage missing data, we performed multiple imputations by chained equations with the fully conditional specification method (56 datasets, 30 iterations). Of the 7,264 observations, 44% were complete. A multilevel imputation model was implemented to take the longitudinal structure of the data into account.^38^ The imputation procedure included all studied exposure and outcome variables. Regression coefficients were pooled using Rubin’s rules. For nonparametric Welch, Wilcoxon–Mann–Whitney, and Kruskal–Wallis tests, however, test statistics were computed separately within each imputed dataset and we used the median of all resulting *P* values.^39^ All statistical analyses were performed with R software (version 4.2.0, Foundation for Statistical Computing, Vienna, Austria).

## Results

### Baseline Characteristics

Patients’ mean age was 67 years and 66% were men (Table 1). Mean eGFR was 34 mL/min/1.73 m^2^, and 70% had albuminuria ≥30 mg/g. Patients were prescribed a median of 7 drug classes per day. In total, 10%-22% reported “being very to extremely bothered” by cramps, itching, fatigue, and stress or worry about kidney disease; a third were classified as having depression. The percentage of patients who reported sleep apnea in the questionnaire and that for those with a diagnosis of sleep apnea in their medical records were 15% and 14%, respectively; 73% of those diagnosed with sleep apnea reported breathing pauses during sleep.

**Table 1:** Patient Characteristics at Baseline (N = 2,765)

Characteristics	Mean ± SD Median (IQR) %	N_missing_
**Sociodemographic Data**		
Age, y	67 ± 13	0
Men	66%	0
Education, y		32
<9	14%	
9 to <12	50%	
≥12	36%	
Occupational status		126
Working	17%	
Retired	73%	
Unemployed	10%	
Living alone	23%	103
**Lifestyle Factors**		
Smoking status		29
Current smoker	11%	
Past smoker	48%	
Never smoker	41%	
Physical activity		171
High	27%	
Moderate	25%	
Low	48%	
**Medical History**		
Diabetes	43%	6
Hypertension	96%	2
Cardiovascular disease	53%	36
Heart failure	13%	6
Other cardiovascular disease^a^	52%	36
Chronic respiratory disease^b^	14%	57
**Measures**		
Body mass index, kg/m^2^	28.7 ± 5.8	29
Obesity (≥ 30 kg/m^2^)	35%	29
Systolic blood pressure (mm Hg)	140 (129-153)	31
Systolic blood pressure ≥ 140 mm Hg	51%	31
Diastolic blood pressure	78 (70-85)	34
Mean blood pressure^c^	98 (91-106)	34
MMSE score	28 (25-29)	23
MMSE score <24	12%	23
eGFR, mL/min/1.73 m^2^	34 ± 13	7
Albumin-to-creatinine ratio (mg/g)	112 (23-521)	332
**Medication**		
Number of prescribed drug classes	7 (4-9)	9
Corticoids	6%	0
Thyroid preparations	12%	0
Long half-life benzodiazepines	4%	0
Antihistamines	3%	0
Beta-blockers	41%	0
**Patient-reported Symptoms**		
Cramps		23
Not at all	28%	
Somewhat	33%	
Moderate	20%	
Very much/extreme	19%	
Itching		34
Not at all	51%	
Somewhat	26%	
Moderate	13%	
Very much/extreme	10%	
Frequency of stress or worries related to kidney disease		74
Not at all	47%	
Somewhat	31%	
Moderate	12%	
Very much/extreme	10%	
Fatigue or exhaustion		37
Not at all	17%	
Somewhat	37%	
Moderate	24%	
Very much/extreme	22%	
**Patient-reported Sleep Apnea**	15%	781
**Depression**		
CES-D score ≥ 10 or antidepressant drug use	33 %	46

Abbreviations: CES-D, Center for Epidemiologic Studies-Depression Scale; eGFR, estimated glomerular filtration rate; IQR, interquartile range; MMSE, Mini-Mental State Examination; SD, standard deviation.

a Other cardiovascular diseases include myocardial infarction, coronary heart disease, stroke or transient ischemic attack, peripheral artery disease, arrhythmias, and cardiac or vascular surgery.

b Chronic obstructive pulmonary disease, asthma, home oxygen use.

c Mean blood pressure = (systolic blood pressure + 2 × diastolic blood pressure)/3.

### Baseline Description of Sleep Duration, Prevalence of Sleep Disturbances, and Trends Over Time

At baseline, the mean 24-hour sleep duration was 7.7 ± 1.7 hours, including 7.2 ± 1.6 hours for nighttime sleep. In total, 16% of the patients were short sleepers (≤6 hours), and 24% were long sleepers (≥9 hours). Thirty-nine percent of patients reported regular and long naps (Fig S1). Daytime sleep tended to be slightly longer at lower eGFR and higher albuminuria (Table S2).

The prevalence of insomnia was 22% (95% CI, 21%-24%), and that of EDS was 15% (95% CI, 14%-16%). Insomnia prevalence decreased with age and was higher in women than men (Table S3).The crude prevalence of EDS tended to be higher at lower eGFR levels, and that of insomnia was slightly higher at higher ACR levels, without gender interaction (Fig 1, Table S3). Both tended to increase as CKD risk category worsened.

**Figure 1:**
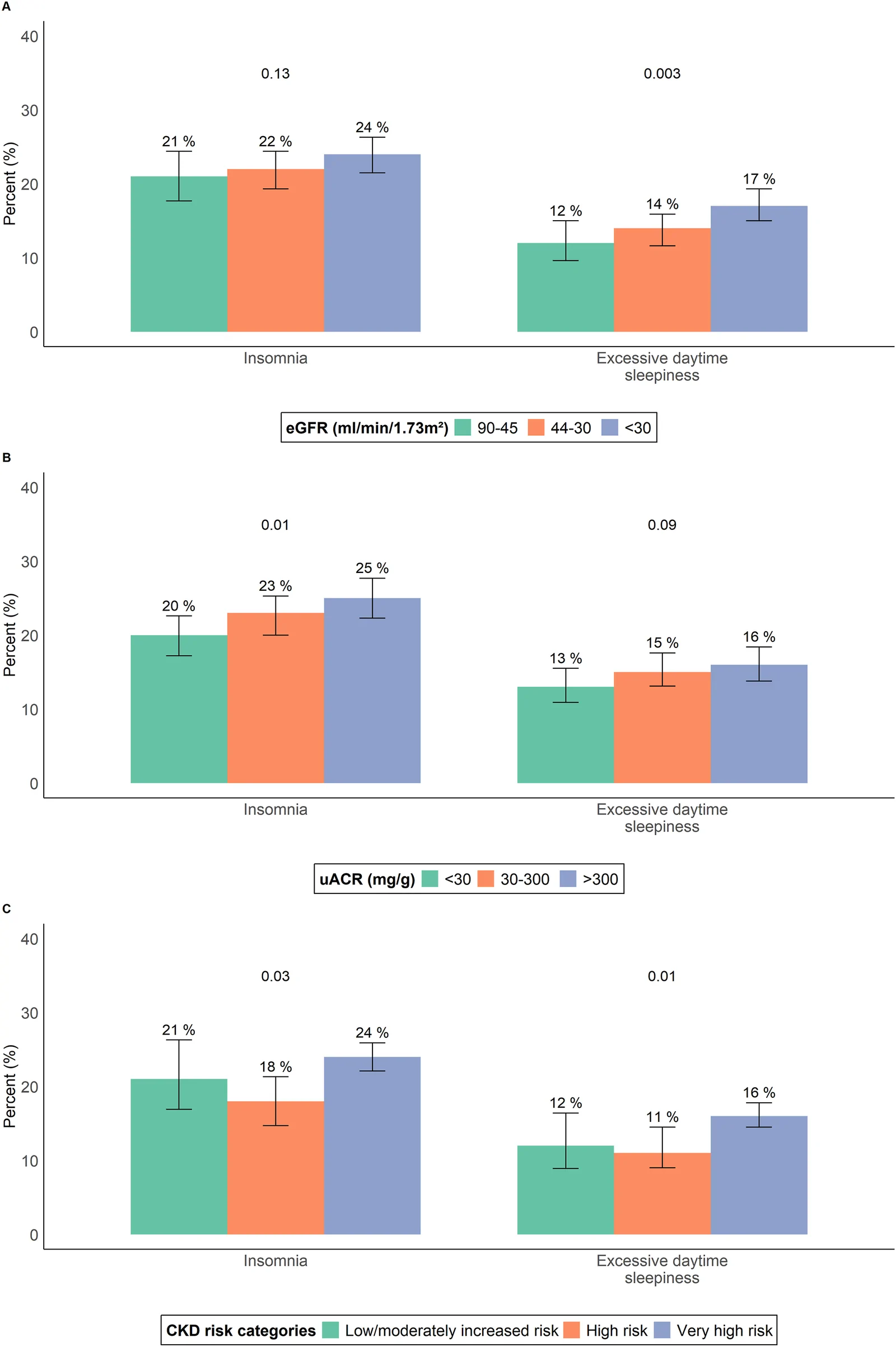
Baseline prevalence of sleep disturbances according to estimated glomerular filtration rate (eGFR), urinary albumin-to-creatinine ratio (uACR), and CKD risk categories.

Although mean eGFR declined by 7.5 mL/min/1.73 m^2^ on average, the crude prevalence of both EDS and insomnia were similar at each time point (Fig 2). When time was modeled continuously, however, insomnia increased slightly with time (OR, 1.06; 95% CI, 1.00-1.11; *P* value, 0.043) per year.

**Figure 2:**
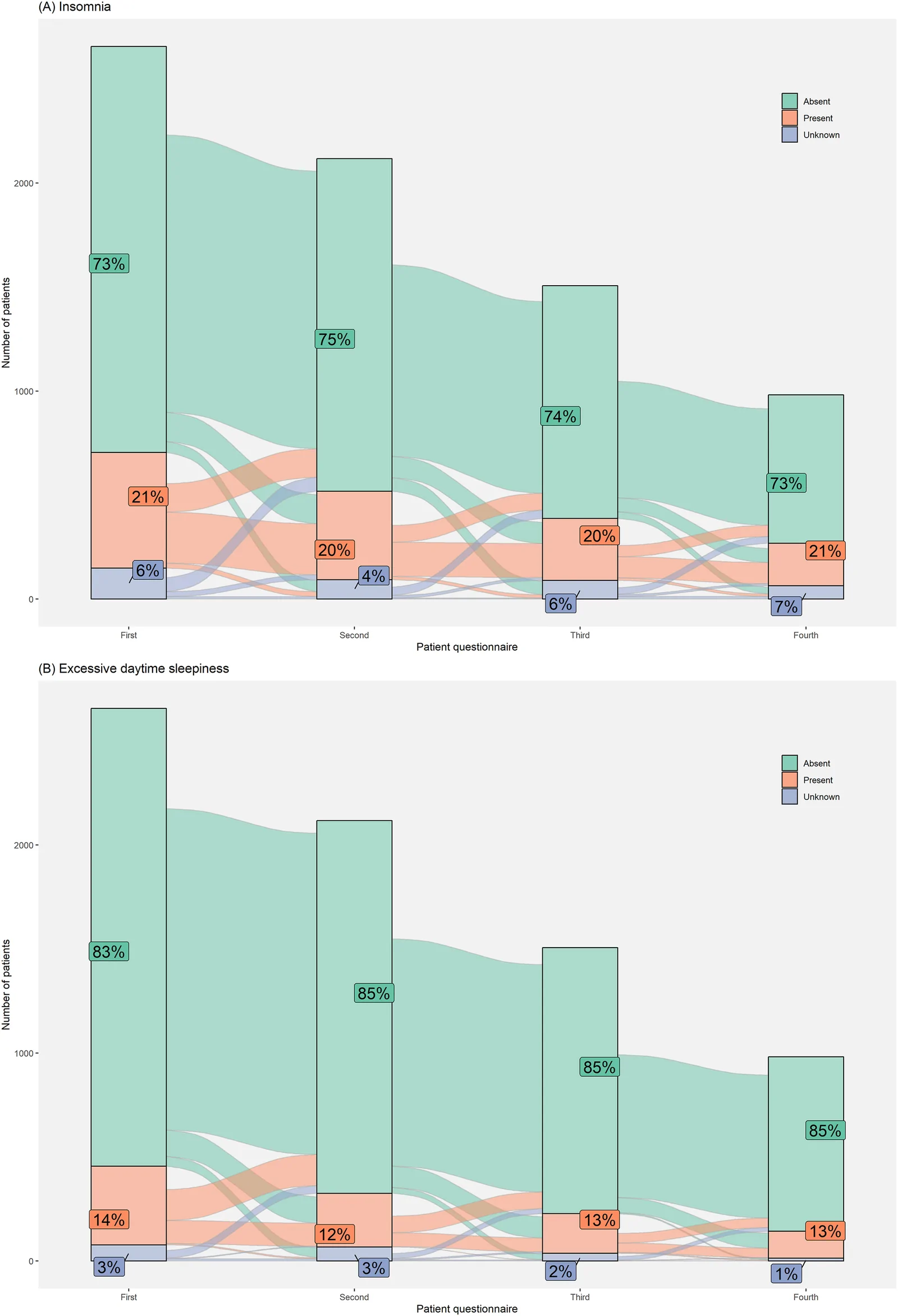
Sankey plots for reported sleep disturbances over 5-year follow-up. (A) Insomnia; (B) Excessive daytime sleep.

### Multivariable Analyses of Factors Associated With of Insomnia

Based on the 7,264 observations collected over 5 years, the 4-variable-adjusted OR of insomnia was not associated with either eGFR and ACR. It was significantly lower with higher age and physical activity and higher in women, in patients living alone or unemployed, those with comorbid conditions, and those prescribed thyroid hormones (Table S4, Model 1). The associations with obesity, diabetes, and chronic respiratory disease, however, were no longer statistically significant after adjustment for other factors, and those with physical activity and thyroid hormones weakened (Table S4, Model 2). In the fully adjusted model, insomnia was strongly related to the severity of cramps, itching, and stress or worry about CKD, independent of age, gender, smoking, heart failure, depression, and sleep apnea (Table 2).

**Table 2:** Multivariable Analysis of Factors Associated With Insomnia (2,765 Patients, 7,264 Observations)^a^

	Fully Adjusted OR (95% CI)^a^^,^^b^	*P* Value
Time since first sleep questionnaire completed (y)	1.07 (1.01-1.13)	0.02
Age (y, per 10-year increase)	0.71 (0.60-0.84)	<0.001
Women vs men	1.77 (1.30-2.42)	<0.001
eGFR (per 10 mL/min/1.73 m^2^ decrease)	0.95 (0.86-1.04)	0.26
ACR in mg/g (per 2-fold increase)	1.03 (0.97-1.09)	0.30
Living alone	1.21 (0.90-1.61)	0.21
Occupational status		0.14
Employed	1	
Retired	1.08 (0.66-1.77)	
Unemployed	1.63 (0.98-2.70)	
Smoking status		0.11
Current smoker	1.33 (0.88-2.02)	
Past smoker	1.34 (1.00-1.78)	
Never smoker	1	
Heart failure	1.61 (1.10-2.36)	0.01
Chronic respiratory disease^c^	1.06 (0.72-1.56)	0.77
Thyroid hormones	1.39 (0.95-2.03)	0.09
Cramps		<0.001^e^
Not at all	1	
Somewhat	1.66 (1.24-2.23)	
Moderate	1.90 (1.37-2.62)	
Very much/extreme	4.66 (3.28-6.62)	
Itching		<0.001^e^
Not at all	1	
Somewhat	1.26 (0.98-1.63)	
Moderate	1.51 (1.11-2.05)	
Very much/Extreme	2.28 (1.60-3.25)	
Frequency of stress or worries related to kidney disease		<0.001^e^
Not at all	1	
Somewhat	1.57 (1.23-2.02)	
Moderate	2.03 (1.46-2.83)	
Very much/extreme	2.30 (1.58-3.36)	
Depression^d^	4.52 (3.53-5.80)	<0.001
Sleep apnea	1.55 (1.14-2.10)	0.005

a OR (95% CI), odds ratio and 95% confidence interval estimated from a mixed effects logistic regression using all 7,264 observations collected over the 5-year follow-up.

b Full multivariable model including time, age, gender, eGFR, log ACR, each social and lifestyle factor, comorbid condition and medication with a *P* value <0.10 in the 2nd multivariable model in Supplementary Table 4, CKD-related symptoms, depression, and sleep apnea.

c Chronic respiratory disease includes chronic obstructive pulmonary disease, asthma, and home oxygen use.

d Depression: Center for Epidemiologic Studies-Depression Scale (CES-D) score ≥ 10 or antidepressant medication.

e *P* value for linear trend; ACR: albumin-to-creatinine ratio in mg/g creatinine; eGFR, estimated glomerular filtration rate.

### Multivariable Analyses of Factors Associated With EDS

The 4-variable-adjusted OR of EDS was higher in women, unemployed patients, and those with comorbid conditions, lower MMSE scores, and prescribed long half-life benzodiazepine and β-blockers; it was lower at higher education and physical activity levels and significantly higher at lower eGFR levels (Table S5, Model 1).The ORs for EDS remained significantly higher with lower eGFR, obesity, heart failure, and benzodiazepine use and lower at a higher physical activity level after adjustment for other social factors, lifestyle, and comorbid conditions (Table S5, Model 2). In the fully adjusted model, EDS had a strong dose–response relation with the severity of fatigue, independent of other significantly associated factors including depression, insomnia, and sleep apnea. In this model, the association of EDS with long half-life benzodiazepine weakened, and those with all other factors, including eGFR, vanished (Table 3).

**Table 3:** Multivariable Analysis of Factors Associated With Excessive Daytime Sleepiness (2765 Patients, 7264 Observations)^a^

	Fully adjusted OR (95% CI)^a^^,^^b^	*P* Value
Age (y) - per 10-year increase	1.13 (0.98-1.32)	0.10
Women vs men	0.81 (0.62-1.07)	0.13
eGFR (per 10 mL/min/1.73 m^2^ decrease)	1.05 (0.96-1.16)	0.30
ACR in mg/g (per 2-fold increase)	0.98 (0.93-1.03)	0.40
Occupational status		0.44
Employed	1	
Retired	1.01 (0.63-1.63)	
Unemployed	1.33 (0.81-2.20)	
Physical activity		0.23^c^
High	0.83 (0.62-1.12)	
Moderate	0.77 (0.59-1.01)	
Low	1	
Obesity (≥30 kg/m^2^)	1.24 (0.96-1.60)	0.10
Heart failure	1.20 (0.85-1.70)	0.31
Long half-life benzodiazepine	1.56 (0.92-2.64)	0.10
Beta-blockers	1.10 (0.86-1.40)	0.46
Frequency of stress or worries related to kidney disease		<0.001^c^
Not at all	1	
Somewhat	1.13 (0.87-1.46)	
Moderate	1.33 (0.95-1.85)	
Very much/extreme	2.11 (1.45-3.06)	
Fatigue or exhaustion		<0.001^c^
Not at all	1	
Somewhat	2.02 (1.29-3.17)	
Moderate	3.59 (2.25-5.74)	
Very much/extreme	13.3 (8.07-22.0)	
Depression^d^	1.58 (1.22-2.05)	<0.001
Insomnia	2.23 (1.73-2.86)	<0.001
Sleep apnea	1.64 (1.22-2.21)	<0.001

Abbreviations: ACR, albumin-to-creatinine ratio in mg/g; CES-D, Center for Epidemiologic Studies-Depression Scale; eGFR, estimated glomerular filtration rate; MMSE, Mini-Mental State Examination.

a OR (95% CI), odds ratio and 95% confidence interval estimated from a mixed effects logistic regression using all 7264 observations collected over the 5-year follow-up.

b Full multivariable model including age, gender, eGFR, log ACR, each social and lifestyle factor, comorbid condition and medication with a *P* value <0.10 in the 2nd multivariable model in Table S5, CKD-related symptoms, depression, insomnia, and sleep apnea (Table S5).

c *P* value for linear trend.

d Depression: CES-D score ≥ 10 or antidepressant medication.

## Discussion

In this large cohort of CKD patients, insomnia and EDS were common, but only EDS was related to decreasing eGFR after controlling for social, lifestyle, and comorbid factors; neither sleep disturbance was associated with albuminuria. Our step-by-step analysis underlines the multifactorial nature of sleep disturbances in CKD, highlighting the contributions of bothersome symptoms and drug-induced effects to their high prevalence, independent of other risk factors. This comprehensive approach offers deeper insight into the underlying determinants of sleep disturbances and their important clinical implications.

To our knowledge, the CKD-REIN study is the first to report such detailed data on insomnia and EDS and their determinants in patients with well-phenotyped CKD. A first important objective of the study was to assess their prevalence and their relation, if any, to eGFR and albuminuria levels, before and after adjustment for age and gender, as well as for common risk factors. Comparing prevalences between studies is, however, challenging because of differences in methods used and the population studied.^17^^,^^40^ In a population-based study, prevalence estimate for insomnia can vary by a factor of 2 to 3, ranging from 8.5%-23.6% depending on whether the criterion of impaired daytime functioning at least 3 times per week is applied.^17^ Our prevalence estimates for insomnia (at least 3 nights/week over the past 3 months) of 30% in women and 19% in men are close to those observed in the French general population at 34% and 25%, respectively, with the same criteria (but over a period of at least 1 month).^41^ Our overall prevalence of 22% (95% CI, 21%-24%) was also close to the pooled estimate of 23% (95% CI, 17%-28%) from a meta-analysis of 13 population-based studies,^8^ but less than half the 48% (95% CI, 30%-67%) from 4 highly heterogenous CKD studies. Insomnia showed no significant dose–effect relation with eGFR or ACR levels after adjustment for age and gender.

Our overall prevalence of EDS—15% (95% CI, 14%-16%)—was somewhat lower than the pooled prevalence of 22% (95% CI, 18%-28%) from 7 small CKD studies using the Epworth Sleepiness Scale,^9^ but in the upper range of those from several population-based studies, ranging from 4%-20.6%.^4^^,^^42^ In contrast to insomnia, however, EDS prevalence tended to increase as eGFR decreased, independent of age, gender, and social, lifestyle, and comorbid factors; this association was no longer statistically significant after adjusting for CKD-related symptoms. In summary, both sleep disturbances appeared common in patients with CKD not receiving dialysis. Although the absence of dose–effect relations with CKD markers does not support a direct role of CKD in the occurrence of insomnia, that observed between EDS and eGFR may suggest they are more closely linked. Of note, 60% of the participants reported a sleep duration within the recommended range of 7-9 hours for optimal health in adults,^1^ and they were more often long sleepers than short sleepers.

The other major objective of this study was to clarify whether or not CKD-related factors might contribute to sleep disturbances independent of other known risk factors. Many factors have been associated with insomnia, including female gender, social and lifestyle factors, sleep apnea, and use of drugs with stimulant effects; the potential impact of (older) age is less consistent.^14^^,^^17^, ^18^, ^19^^,^^41^^,^^43^ Insomnia is also strongly associated with anxiety and depression and was once considered one of their symptoms.^16^^,^^44^ Studies in patients receiving dialysis have highlighted the disruptive effect of the dialysis session shift time on sleep patterns and the link between symptom burden and high insomnia prevalence in this population.^45^ In CKD-REIN, insomnia was strongly associated with the severity of cramps, itching, and stress, independent of other known factors. These findings suggest that these commonly reported symptoms contribute to insomnia in CKD^7^^,^^24^; cramps are potentially related to use of lipid-lowering drugs.^46^ Thyroid hormone use was also associated with increased insomnia, although model adjustment mitigated this relation. The narrow therapeutic window of these hormones may result in episodes of hyperthyroidism, potentially causing insomnia or disrupting sleep.

Less is known about risk factors for EDS.^4^^,^^13^^,^^47^^,^^48^ In most studies, its prevalence is similar in men and women.^42^^,^^49^^,^^50^ It might result from sleep fragmentation because of insomnia or sleep apnea as well as from mood or cognitive disorders or iatrogenic effects of medications.^16^^,^^37^ It is more common in individuals with obesity, diabetes, and cardiorespiratory diseases^4^^,^^42^^,^^50^^,^^51^ and may regress with less use of hypnotics and decreased insomnia and depressive symptoms.^51^ Although several of these comorbid conditions and cognitive impairment were significantly associated here with increased EDS, independent of age, gender, and CKD markers, only female gender, lower eGFR, obesity, low physical activity, and long half-life benzodiazepine use remained so after adjusting for each other. However, none of these associations remained significant in the fully adjusted model. This study suggests that EDS in CKD patients is mainly linked to fatigue, the most frequent symptom and the most strongly related to eGFR decline, independent of mood and other sleep disturbances.^24^ Although EDS and fatigue are strongly associated, it is worth noting that they refer to different perceived feelings, as evidenced by the incomplete overlap of complaints about them. CKD metabolic complications, uremic toxins, and systemic inflammation may be some of the underlying factors explaining the coexistence of sleep disturbances and fatigue, in CKD.^27^^,^^52^

Major strengths of this study include the large cohort size with repeated assessments over 5 years, the representativeness of nephrology clinics, the use of standardized questions for sleep disturbance assessment, and the high response rates to the patient questionnaires. Our comprehensive data collection, including CKD markers and symptoms, social and lifestyle factors, comorbid conditions, and prescribed drugs, is unique in this field and allowed us to investigate a wide range of potential determinants with high study power.

This study also has limitations. First, the self-reporting of sleep concerns could have resulted in misclassification of the sleep disturbances. Self-administered questionnaires have been shown to have good to very good validity compared with face-to face interview for assessing insomnia symptoms, and we used recommended standard criteria to define insomnia.^16^^,^^37^ In contrast, for EDS, reliance on selected questionnaire items rather than validated diagnostic instruments such as the Epworth scale may have resulted in potential misclassification and underestimation of its prevalence compared with studies using this instrument.^4^^,^^9^^,^^42^ Likewise, it is well-established that sleep questionnaire-based studies substantially underestimate the true prevalence of sleep apnea, compared with those using instrumental monitoring devices, as shown in a recent meta-analysis of CKD studies reporting this prevalence to be twice as low in the former than the latter (33% [16%-49%] vs 66% [52%-72%]).^53^ Our prevalence estimate of 14% is low and appears to primarily reflect patients’ awareness of their sleep apnea diagnosis; 73% of the study participants with a diagnosis in their medical record reported breathing pauses during sleep. Second, although the CKD-REIN study is longitudinal with repeated assessments, the analytic approach evaluates concurrent associations between sleep disturbances and exposures updated at the time of completion of the self-administered questionnaire and does not allow the timing of these associations to be established. This limitation is particularly important for dynamic variables such as fatigue and depression, which may both influence and be influenced by sleep disturbances. This design is nonetheless appropriate when the disturbance studied remains stable during follow-up, and the studied factors are more likely to contribute to than result from sleep disturbances. Third, although confounding by indication is often discussed in medication association studies, it is unlikely here because the drugs studied were selected for their potential adverse effects and not their indications for the outcomes studied. Finally, in contrast with other studied variables, the MMSE was administered at baseline only, which may have underestimated the relation between cognitive impairment and sleep disturbances.

Despite the study limitations, these findings have important clinical implications for improving patient well-being and health. They encourage clinicians to evaluate nonpharmacologic contributors, eg, cramps, itching, stress, and fatigue, and to review medications that may impair sleep in the population of CKD patients. Second, treatment strategy depends on the type of sleep disorder, its severity, and identified underlying cause(s). For insomnia, this study highlights the need to reassess the choice of medications and their dosage to avoid potential side effects (eg, thyroid hormones and statins), and treat bothersome symptoms, particularly pruritus.^54^ Physical activity may also be useful as a potential nondrug intervention^55^ and treating insomnia necessary, but evidence-based guidelines are lacking.^16^^,^^56^ For EDS, prioritizing short rather than long half-life classes in benzodiazepine users may help avoid residual daytime effect. CKD-related fatigue, which was shown to strongly associate with EDS, may be less easily modifiable.

In conclusion, this study challenges the assumption that insomnia and EDS in patients with CKD not receiving dialysis are driven solely by declining kidney function. Instead, it shows a more nuanced picture that highlights the multifactorial nature of sleep disturbances in CKD, with the contributions of bothersome symptoms, comorbid conditions, and drug-induced effects to their high prevalence. Addressing these sleep disruptions through targeted therapies, aimed at reducing pruritis, cramps, and stress, and lifestyle modifications may improve well-being and daily functioning, but whether this can mitigate long-term CKD outcomes remains to be determined.

## References

[1] Watson N.F., Badr M.S., Consensus Conference Panel (2015). Joint consensus statement of the American Academy of Sleep Medicine and Sleep Research Society on the recommended amount of sleep for a healthy adult: methodology and discussion. J Clin Sleep Med.

[2] Benz F., Meneo D., Baglioni C., Hertenstein E. (2023). Insomnia symptoms as risk factor for somatic disorders: An umbrella review of systematic reviews and meta-analyses. J Sleep Res.

[3] Fernandez-Mendoza J. (2025). Insomnia phenotypes, cardiovascular risk and their link to brain health. Circ Res.

[4] Pérez-Carbonell L., Mignot E., Leschziner G., Dauvilliers Y. (2022). Understanding and approaching excessive daytime sleepiness. Lancet.

[5] Bock J., Covassin N., Somers V. (2022). Excessive daytime sleepiness: an emerging marker of cardiovascular risk. Heart.

[6] Wennberg A., Wu M., Rosenberg P., Spira A. (2017). Sleep disturbance, cognitive decline, and dementia: a review. Semin Neurol.

[7] Fletcher B.R., Damery S., Aiyegbusi O.L. (2022). Symptom burden and health-related quality of life in chronic kidney disease: a global systematic review and meta-analysis. PLOS Med.

[8] Tan L.H., Chen P.S., Chiang H.Y. (2022). Insomnia and poor sleep in CKD: a systematic review and meta-analysis. Kidney Med.

[9] Huang Z., Tang X., Zhang T., Qiu S., Xia Z., Fu P. (2019). Prevalence of sleep apnoea in non-dialysis chronic kidney disease patients: a systematic review and meta-analysis. Nephrology.

[10] Vendeville N., Mucsi I., Molnar M.Z. (2025). Sleep-related disorders in patients with chronic kidney disease and kidney transplant recipients. Clin J Am Soc Nephrol.

[11] Ricardo A.C., Knutson K., Chen J. (2017). The association of sleep duration and quality with CKD progression. J Am Soc Nephrol.

[12] Turek N.F., Ricardo A.C., Lash J.P. (2012). Sleep disturbances as nontraditional risk factors for development and progression of CKD: review of the evidence. Am J Kidney Dis.

[13] Jaussent I., Cristol J.P., Stengel B. (2016). Impact of sleep disturbances on kidney function decline in the elderly. Eur Respir J.

[14] Lyons O.D. (2024). Sleep disorders in chronic kidney disease. Nat Rev Nephrol.

[15] Gopal A., Farragher J., Jassal S.V., Mucsi I. (2025). Sleep disorders in CKD: a review. Am J Kidney Dis.

[16] Riemann D., Baglioni C., Bassetti C. (2017). European guideline for the diagnosis and treatment of insomnia. J Sleep Res.

[17] Porcheret K., Hopstock L.A., Nilsen K.B. (2024). Prevalence of insomnia in a general adult population cohort using different diagnostic criteria: the seventh survey of the Tromsø study 2015–2016. Sleep Med.

[18] Morin C.M., Jarrin D.C. (2022). Epidemiology of insomnia: prevalence, course, risk factors, and public health burden. Sleep Med Clin.

[19] Zeng L.N., Zong Q.Q., Yang Y. (2020). Gender difference in the prevalence of insomnia: a meta-analysis of observational studies. Front Psychiatry.

[20] Plantinga L., Lee K., Inker L.A. (2011). Association of sleep-related problems with CKD in the United States, 2005-2008. Am J Kidney Dis.

[21] Ogna A., Ogna V.F., Rubio J.H. (2016). Sleep characteristics in early stages of Chronic kidney disease in the HypnoLaus cohort. Sleep.

[22] Koh J.H., Lim C.Y.J., Yam K.J.M. (2024). Bidirectional association of sleep disorders with chronic kidney disease: a systematic review and meta-analysis. Clin Kidney J.

[23] Chen J., Ricardo A.C., Reid K.J. (2022). Sleep, cardiovascular risk factors, and kidney function: The Multi-Ethnic Study of Atherosclerosis (MESA). Sleep Health.

[24] Speyer E., Tu C., Zee J. (2024). Symptom burden and its impact on quality of life in patients with moderate to severe CKD: The International Chronic Kidney Disease Outcomes and Practice Patterns Study (CKDopps). Am J Kidney Dis.

[25] Faucon A.L., Lambert O., Massy Z. (2024). Sex and the risk of atheromatous and nonatheromatous cardiovascular disease in CKD: findings from the CKD-REIN Cohort Study. Am J Kidney Dis.

[26] Viggiano D., Wagner C.A., Martino G. (2020). Mechanisms of cognitive dysfunction in CKD. Nat Rev Nephrol.

[27] Wagner C.A., Massy Z.A., Capasso G. (2025). Translational research on cognitive impairment in chronic kidney disease. Nephrol Dial Transplant.

[28] Stengel B., Metzger M., Combe C. (2019). Risk profile, quality of life and care of patients with moderate and advanced CKD: the French CKD-REIN Cohort Study. Nephrol Dial Transplant.

[29] Stengel B., Combe C., Jacquelinet C. (2014). The French Chronic Kidney Disease-Renal Epidemiology and Information Network (CKD-REIN) cohort study. Nephrol Dial Transplant.

[30] Bull F.C., Maslin T.S., Armstrong T. (2009). Global Physical Activity Questionnaire (GPAQ): nine country reliability and validity study. J Phys Act Health.

[31] World Health Organization Collaborating Centre for Drug Statistics Methodology (2016). ATCDDD - ATC/DDD Index. http://www.whocc.no/atc_ddd_index.

[32] Levey A.S., Stevens L.A., Schmid C.H. (2009). A New equation to estimate glomerular filtration rate. Ann Intern Med.

[33] Sumida K., Nadkarni G.N., Grams M.E. (2020). Conversion of urine protein–creatinine ratio or urine dipstick protein to urine albumin–creatinine ratio for use in chronic kidney disease screening and prognosis: an individual participant–based meta-analysis. Ann Intern Med.

[34] Folstein M.F., Folstein S.E., McHugh P.R. (1975). Mini-mental state. J Psychiatr Res.

[35] Hays R.D., Kallich J.D., Mapes D.L., Coons S.J., Carter W.B. (1994). Development of the Kidney Disease Quality of Life (KDQOLTM) instrument. Qual Life Res.

[36] Kohout F.J., Berkman L.F., Evans D.A., Cornoni-Huntley J. (1993). Two shorter forms of the CES-D (Center for Epidemiological Studies Depression) depression symptoms index. J Aging Health.

[37] Partinen M. (1995). Basic Nordic Sleep Questionnaire (BNSQ): a quantitated measure of subjective sleep complaints. J Sleep Res.

[38] van Buuren S. (2018).

[39] Austin P.C., Eekhout I., van Buuren (2024). Evaluating the median *p*-value method for assessing the statistical significance of tests when using multiple imputation. J Appl Stat.

[40] Chung K.F., Yeung W.F., Ho F.Y.Y., Yung K.P., Yu Y.M., Kwok C.W. (2015). Cross-cultural and comparative epidemiology of insomnia: the Diagnostic and Statistical Manual (DSM), International Classification of Diseases (ICD) and International Classification of Sleep Disorders (ICSD). Sleep Med.

[41] Leger D., Guilleminault C., Dreyfus J.P., Delahaye C., Paillard M. (2000). Prevalence of insomnia in a survey of 12 778 adults in France. J Sleep Res.

[42] Ohayon M.M. (2008). From wakefulness to excessive sleepiness: what we know and still need to know. Sleep Med Rev.

[43] Ong J.C., Crawford M.R. (2013). Insomnia and obstructive sleep apnea. Sleep Med Clin.

[44] Alvaro P.K., Roberts R.M., Harris J.K. (2013). A systematic review assessing bidirectionality between sleep disturbances, anxiety, and depression. Sleep.

[45] Cheng E., Evangelidis N., Guha C. (2021). Patient experiences of sleep in dialysis: systematic review of qualitative studies. Sleep Med.

[46] Laville S.M., Gras-Champel V., Hamroun A. (2024). Kidney function decline and serious adverse drug reactions in patients with CKD. Am J Kidney Dis.

[47] Joo S., Baik I., Yi H., Jung K., Kim J., Shin C. (2009). Prevalence of excessive daytime sleepiness and associated factors in the adult population of Korea. Sleep Med.

[48] Kolla B.P., He J.P., Mansukhani M.P., Frye M.A., Merikangas K. (2020). Excessive sleepiness and associated symptoms in the U.S. adult population: prevalence, correlates, and comorbidity. Sleep Health.

[49] Baldwin C.M., Kapur V.K., Holberg C.J., Rosen C., Nieto F.J. (2004). Associations between gender and measures of daytime somnolence in the sleep heart health study. Sleep.

[50] Hayley A.C., Williams L.J., Kennedy G.A., Berk M., Brennan S.L., Pasco J.A. (2014). Prevalence of excessive daytime sleepiness in a sample of the Australian adult population. Sleep Med.

[51] Jaussent I., Morin C.M., Ivers H., Dauvilliers Y. (2020). Natural history of excessive daytime sleepiness: a population-based 5-year longitudinal study. Sleep.

[52] Massy Z.A., Chesnaye N.C., Larabi I.A. (2022). The relationship between uremic toxins and symptoms in older men and women with advanced chronic kidney disease. Clin Kidney J.

[53] Pisano A., Zoccali C., Bolignano D., D’Arrigo G., Mallamaci F. (2024). Sleep apnoea syndrome prevalence in chronic kidney disease and end-stage kidney disease patients: a systematic review and meta-analysis. Clin Kidney J.

[54] Metzger M., Abdel-Rahman E.M., Boykin H., Song M.K. (2021). A narrative review of management strategies for common symptoms in advanced CKD. Kidney Int Rep.

[55] Riedel A., Benz F., Deibert P. (2024). The effect of physical exercise interventions on insomnia: A systematic review and meta-analysis. Sleep Med Rev.

[56] Nalankilli K., Stallworthy E., Ducharlet K. (2025). Treatment of insomnia, restless legs, cramps, and pain associated with chronic kidney disease: results from a multinational survey of kidney supportive care practice. BMC Nephrol.

