# Supplementary material for: Sleep Disturbances in Chronic Kidney Disease – The CKD-REIN Cohort Study
Source: Kidney Med. 2026 Sep 15;8(11):101530. doi: 10.1016/j.xkme.2026.101530 (PMC13679367; doi:10.1016/j.xkme.2026.101530)
Supplement: Supplementary file 1 [file mmc1.pdf]

Supplementary Material
Table S1 – List and baseline description of prescribed potentially sedative or stimulant medications studied

	ATC code	N (%)
**Corticoids**		**174 (6 %)**
Fludrocortisone	ATC-H02AA02	3
Betamethasone	ATC-H02AB01	0
Dexamethasone	ATC-H02AB02	4
Methylprednisolone	ATC-H02AB04	4
Prednisolone	ATC-H02AB06	39
Prednisone	ATC-H02AB07	127
Triamcinolone	ATC-H02AB08	1
**Thyroid preparations**		**328 (12 %)**
Levothyroxine sodium	ATC-H03AA01	326
Liothyronine sodium	ATC-H03AA02	1
Combinations of levothyroxine and liothyronine	ATC-H03AA03	2
Tiratricol	ATC-H03AA04	0
**Long half-life benzodiazepines**		**121 (4 %)**
Clonazepam	ATC-N03AE01	16
Diazepam	ATC-N05BA01	1
Potassium clorazepate	ATC-N05BA05	15
Bromazepam	ATC-N05BA08	64
Prazepam	ATC-N05BA11	21
Nordazepam	ATC-N05BA16	2
Ethyl loflazepate	ATC-N05BA18	0
Clobazam	ATC-N05BA09	2
Nitrazepam	ATC-N05CD02	2
Flunitrazepam	ATC-N05CD03	0
**Antihistamines**		**92 (3 %)**
Diphenhydramine	ATC-R06AA02	0
Doxylamine	ATC-R06AA09	0
Dimenhydrinate	ATC-R06AA11	0
Doxylamine, combinations	ATC-R06AA59	0
Dimenhydrinate, combinations	ATC-R06AA61	0
Brompheniramine	ATC-R06AB01	0
Dexchlorpheniramine	ATC-R06AB02	1
Dexchlorpheniramine, combinations	ATC-R06AB52	0
Alimemazine	ATC-R06AD01	3
Promethazine	ATC-R06AD02	0
Mequitazine	ATC-R06AD07	1
Oxomemazine	ATC-R06AD08	1
Promethazine, combinations	ATC-R06AD52	0
Meclozine	ATC-R06AE05	0
Cetirizine	ATC-R06AE07	11
Levocetirizine	ATC-R06AE09	16
Cyproheptadine	ATC-R06AX02	0
Loratadine	ATC-R06AX13	6
Ketotifen	ATC-R06AX17	0
Ebastine	ATC-R06AX22	9
Mizolastine	ATC-R06AX25	1
Fexofenadine	ATC-R06AX26	0
Desloratadine	ATC-R06AX27	35
Rupatadine	ATC-R06AX28	5

Bilastine	ATC-R06AX29	4
**Beta-blockers**		**1137 (41 %)**
Pindolol	ATC-C07AA03	3
Propranolol	ATC-C07AA05	26
Timolol	ATC-C07AA06	0
Nadolol	ATC-C07AA12	11
Tertatolol	ATC-C07AA16	2
Metoprolol	ATC-C07AB02	33
Atenolol	ATC-C07AB03	147
Acebutolol	ATC-C07AB04	51
Betaxolol	ATC-C07AB05	14
Bisoprolol	ATC-C07AB07	508
Celiprolol	ATC-C07AB08	30
Nebivolol	ATC-C07AB12	265
Labetolol	ATC-C07AG01	15
Metoprolol and thiazides	ATC-C07BB02	0
Bisoprolol and thiazides	ATC-C07BB07	8
Nebivolol and thiazides	ATC-C07BB12	7
Atenolol and other diuretics	ATC-C07CB03	0
Timolol, thiazides and other diuretics	ATC-C07DA06	0
Metoprolol and felodipine	ATC-C07FB02	12
Atenolol and nifedipine	ATC-C07FB03	12

Table S2 –Description of sleep duration by gender, age, estimated glomerular filtration rate (eGFR), and albuminuria level at baseline (N= 2765, imputed data)

	N	24-hour sleep duration (hours) Mean±SD	Nighttime sleep duration (hours) Mean±SD	Daytime sleep duration (min)* Median (IQR)	N**	Daytime sleep duration (min)** Median (IQR)
All	2765	7.7 ± 1.68	7.2 ± 1.57	30(10-60)	2108	60(30-75)
Gender						
Women	951	7.5 ± 1.74	7.1 ± 1.64	30(0-60)	653	60(30-60)
Men	1814	8 ± 1.74	7.3 ± 1.53	30(15-60)	1455	60(30-75)
p		***<0.001*[1]**	***<0.001*[1]**	***<0.001*[4]**		*0.19*[4]
Age (years)						
< 65	920	7.4 ± 1.63	7.1 ± 1.55	30(0-60)	663	60(30-75)
65-75	982	7.7 ± 1.69	7.3 ± 1.57	30(10-60)	752	60(30-60)
≥ 75	863	7.9 ± 1.69	7.3 ± 1.57	35(15-60)	693	60(30-90)
p		***<0.001*[2]**	***0.014*[2]**	***<0.001*[3]**		*0.17*[3]
CKD-EPI eGFR (mL/min/1.73 m²)						
≥ 45	557	7.6 ± 1.57	7.2 ± 1.45	30(0-60)	403	45(30-60)
30-44	1005	7.6 ± 1.74	7.2 ± 1.61	30(3-60)	757	60(30-63)
< 30	1203	7.8 ± 1.68	7.3 ± 1.59	40(15-60)	948	60(30-90)
p		*0.06*[2]	*0.44*[3]	***<0.001*[3]**		***0.001*[3]**
Albumin-to-creatinine ratio (mg/g)						
< 30	821	7.6 ± 1.57	7.2 ± 1.48	30(0-60)	592	45(30-60)
30-299	971	7.7 ± 1.69	7.2 ± 1.57	30(10-60)	752	60(30-90)
≥ 300	973	7.7 ± 1.76	7.3 ± 1.64	40(15-60)	764	60(30-90)
p		*0.20*[2]	*0.33*[3]	***<0.001*[3]**		***<0.001*[3]**

* Analysis based on the overall population including patients who did not report naps
** Analysis restricted to patients who reported taking naps.
[1]Welch test – median P over the 56 imputed data sets
[2]ANOVA
[3]Kruskal-Wallis test – median P over the 56 imputed data sets
[4]Wilcoxon/Mann-Whitney test – median P over the 56 imputed data sets

Table S3 – Description of sleep disturbances by gender, age, estimated glomerular filtration rate (eGFR), albuminuria level and CKD risk category at baseline (N= 2765, imputed data)

	N	Insomnia	Excessive daytime sleepiness	Sleep apnea
All	2765	22 %	15 %	15 %
Sex				
Women	951	30 %	17 %	11 %
Men	1814	19 %	14 %	17 %
*P-value**		***<0.001***	***0.02***	***<0.001***
Age				
< 65	920	29%	15%	13%
65-75	982	21%	13%	18%
≥ 75	863	17%	16%	15%
P for trend[1]		***<0.001***	*0.87*	*0.15*
CKD-EPI eGFR (mL/min/1.73 m^2)				
≥45	557	21 %	12 %	14 %
30-45	1005	22 %	14 %	15 %
<30	1203	24 %	17 %	16 %
P for trend[1]		*0.13*	***0.003***	*0.44*
Albumin-to-creatinine ratio (mg/g)				
<30	821	20 %	13 %	14 %
30-299	971	23 %	15 %	14 %
≥ 300	973	25 %	16 %	18 %
P for trend[1]		***0.01***	*0.09*	***0.03***
CKD risk categories[2]				
Low or moderately increased risk	288	21%	12%	14%
High risk	524	18%	11%	13%
Very high risk	1953	24%	16%	16%
P for trend[1]		***0.03***	***0.01***	*0.13*

*Chi2 test

[1] median P for trend over the 56 imputed data sets.

[2] KDIGO CKD risk categories: low or moderately increased risk, (ACR<30 and eGFR≥45) or (ACR, 30-300 and eGFR≥ 60); high risk ,(ACR<30 and 30 ≤eGFR < 45) or (ACR, 30-300 and 45 ≤eGFR < 60) or (ACR>300 and eGFR ≥ 60); very high risk ,(ACR<30 and eGFR < 30) or (ACR, 30-300 and eGFR < 45) or (ACR>300 and eGFR < 60)

Kidney Disease: Improving Global Outcomes (KDIGO) CKD Work Group. KDIGO 2024 Clinical Practice Guideline for the Evaluation and Management of Chronic Kidney Disease. Kidney Int. 2024;105(4S):S117–S314.

Table S4 – First and second multivariable analyses for insomnia (2765 patients, 7264 observations, imputed data)

	Model 1[1]		Model 2[2]	
	**Adjusted OR (95% CI)**	**P**	**Adjusted OR (95% CI)**	**P**
Time since first sleep questionnaire completed (years)	**1.07 (1.02-1.13)**	**0.01**	**1.09 (1.03-1.15)**	**0.003**
Age (years) - per 10-year increase	**0.72 (0.64-0.82)**	**<0.001**	**0.73 (0.62-0.87)**	**<0.001**
Women *vs* Men	**3.19 (2.32-4.38)**	**<0.001**	**2.84 (2.01-4.00)**	**<0.001**
eGFR (per 10 ml/min/1.73m² decrease)	1.08 (0.97-1.20)	0.16	1.06 (0.96-1.17)	0.27
ACR in mg/g (per 2-fold increase)	1.05 (1.00-1.11)	0.07	1.04 (0.98-1.10)	0.16
Living alone	**1.51 (1.11-2.07)**	**0.01**	**1.49 (1.09-2.03)**	**0.01**
Occupational status		**0.002**		**0.01**
Employed	1		1	
Retired	1.52 (0.88-2.65)		1.30 (0.75-2.25)	
Unemployed	**2.82 (1.58-5.01)**		**2.39 (1.35-4.23)**	
Education (years)		0.78*		
< 9	1			
9 - < 12	0.93 (0.59-1.46)			
≥ 12	0.93 (0.58-1.51)			
Smoking status		0.06		0.10
Current smoker	1.58 (1.01-2.46)		1.48 (0.95-2.30)	
Past smoker	1.36 (0.99-1.86)		1.32 (0.97-1.81)	
Never smoker	1		1	
Physical activity		**0.04***		0.11*
Low	1		1	
Moderate	**0.76 (0.58-1.00)**		0.79 (0.60-1.04)	
High	**0.74 (0.55-0.98)**		0.79 (0.59-1.05)	
Obesity (≥ 30 kg/m²)	**1.41 (1.06-1.87)**	**0.02**	1.22 (0.90-1.64)	0.20
Diabetes	**1.48 (1.10-1.99)**	**0.01**	1.20 (0.88-1.65)	0.24
Heart failure	**2.04 (1.35-3.10)**	**<0.001**	**1.77 (1.17-2.69)**	**0.007**
Chronic respiratory disease	**1.83 (1.19-2.80)**	**0.01**	1.48 (0.97-2.27)	0.07
Systolic blood pressure ≥ 140 mm Hg	0.83 (0.66-1.05)	0.12		
Baseline MMSE score <24	0.96 (0.60-1.55)	0.88		
Thyroid hormones	1.50 (0.99-2.26)	0.05	1.43 (0.95-2.16)	0.08
Corticoids	1.26 (0.77-2.07)	0.36		
Cramps		**<0.001***		
Not at all	1			
Somewhat	**1.94 (1.45-2.61)**			
Moderate	**2.68 (1.94-3.71)**			
Very much/Extreme	**8.52 (5.98-12.1)**			
Itching		**<0.001***		
Not at all	1			
Somewhat	**1.56 (1.22-2.01)**			
Moderate	**2.52 (1.86-3.43)**			
Very much/Extreme	**4.92 (3.44-7.03)**			
Frequency of stress or worries related to kidney disease		**<0.001***		
Not at all	1			
Somewhat	**2.06 (1.60-2.64)**			
Moderate	**3.83 (2.75-5.33)**			
Very much/Extreme	**7.12 (4.86-10.4)**			
Depression**	**7.24 (5.67-9.25)**	**<0.001**		
Sleep apnea	**2.27 (1.64-3.13)**	**<0.001**		

ACR: albumin to creatinine ratio; eGFR, estimated glomerular filtration rate; CES-D, Center for Epidemiologic Studies-Depression Scale; MMSE: mini mental state examination; OR (95% CI), odds ratio and 95% confidence interval;
*P-value for linear trend; **Depression: CES-D score ≥ 10 or antidepressant medication
[1] First model: OR for each factor adjusted for time, age, gender, eGFR and ACR.
[2] Second model including time, age, gender, eGFR, ACR, as well as social and lifestyle factors, comorbidities and medications with a p-value <0.10 in the first model.

Table S5 – First and second multivariable analyses for excessive daytime sleepiness (2765 patients, 7264 observations, imputed data)

	Model 1[1]		Model 2[2]	
	Adjusted OR (95% CI)	P	Adjusted OR (95% CI)	P
Age (years) - per 10-year increase	1.04 (0.94-1.16)	0.46	1.00 (0.86-1.17)	0.99
Women *vs* Men	**1.59 (1.20-2.09)**	**<0.001**	1.32 (0.99-1.75)	0.06
eGFR (per 10 ml/min/1.73m² decrease)	**1.19 (1.08-1.32)**	**<0.001**	**1.16 (1.05-1.28)**	**<0.001**
ACR in mg/g (per 2-fold increase)	1.01 (0.96-1.06)	0.63	1.00 (0.95-1.06)	0.92
Occupational status		**0.007**		**0.03**
Employed	1		1	
Retired	1.31 (0.80-2.14)		1.04 (0.64-1.72)	
Unemployed	**2.31 (1.36-3.90)**		**1.87 (1.11-3.17)**	
Education (years)		**0.004***		0.19*
< 9	1		1	
9 - < 12	**0.59 (0.40-0.87)**		0.68 (0.45-1.03)	
≥ 12	**0.55 (0.36-0.82)**		0.74 (0.48-1.16)	
Smoking status		0.14		
Current smoker	1.22 (0.80-1.87)			
Past smoker	1.33 (1.00-1.77)			
Never smoker	1			
Physical activity		**<0.001***		**0.003***
High	**0.58 (0.43-0.78)**		**0.64 (0.48-0.86)**	
Moderate	**0.57 (0.43-0.74)**		**0.61 (0.47-0.80)**	
Low	1		1	
Obesity (≥ 30 kg/m²)	**1.72 (1.34-2.22)**	**<0.001**	**1.44 (1.10-1.89)**	**0.01**
Diabetes	**1.48 (1.13-1.92)**	**<0.001**	1.12 (0.84-1.48)	0.43
Heart failure	**1.93 (1.35-2.76)**	**<0.001**	**1.61 (1.12-2.32)**	**0.01**
Chronic respiratory disease	**1.50 (1.03-2.19)**	**0.03**	1.25 (0.86-1.82)	0.24
Baseline MMSE < 24	**1.68 (1.13-2.51)**	**0.01**	1.16 (0.75-1.78)	0.51
Long half-life benzodiazepine	**2.83 (1.64-4.90)**	**<0.001**	**2.58 (1.49-4.47)**	**<0.001**
Antihistamines	1.49 (0.83-2.69)	0.18		
Beta-blockers	**1.32 (1.03-1.70)**	**0.03**	1.15 (0.89-1.48)	0.29
Frequency of stress or worries related to kidney disease		**<0.001***		
Not at all	1			
Somewhat	**1.59 (1.23-2.05)**			
Moderate	**3.02 (2.19-4.17)**			
Very much/Extreme	**7.87 (5.47-11.3)**			
Fatigue or exhaustion		**<0.001***		
Not at all	1			
Somewhat	**2.44 (1.57-3.79)**			
Moderate	**5.46 (3.47-8.61)**			
Very much/Extreme	**31.1 (19.3-50.3)**			
Depression**	**4.79 (3.80-6.03)**	**<0.001**		
Insomnia	**4.43 (3.48-5.64)**	**<0.001**		
Sleep apnea	**2.77 (2.05-3.74)**	**<0.001**		

ACR: albumin to creatinine ratio; eGFR, estimated glomerular filtration rate; CES-D, Center for Epidemiologic Studies-Depression Scale; MMSE: mini mental state examination; OR (95% CI), odds ratio and 95% confidence interval;
*P-value for linear trend; **Depression: CES-D score ≥ 10 or antidepressant medication
[1] First model: OR for each factor adjusted for age, gender, eGFR and ACR.
[2] Second model including age, gender, eGFR, ACR, as well as social and lifestyle factors, comorbidities and medications with a p-value <0.10 in the first model.

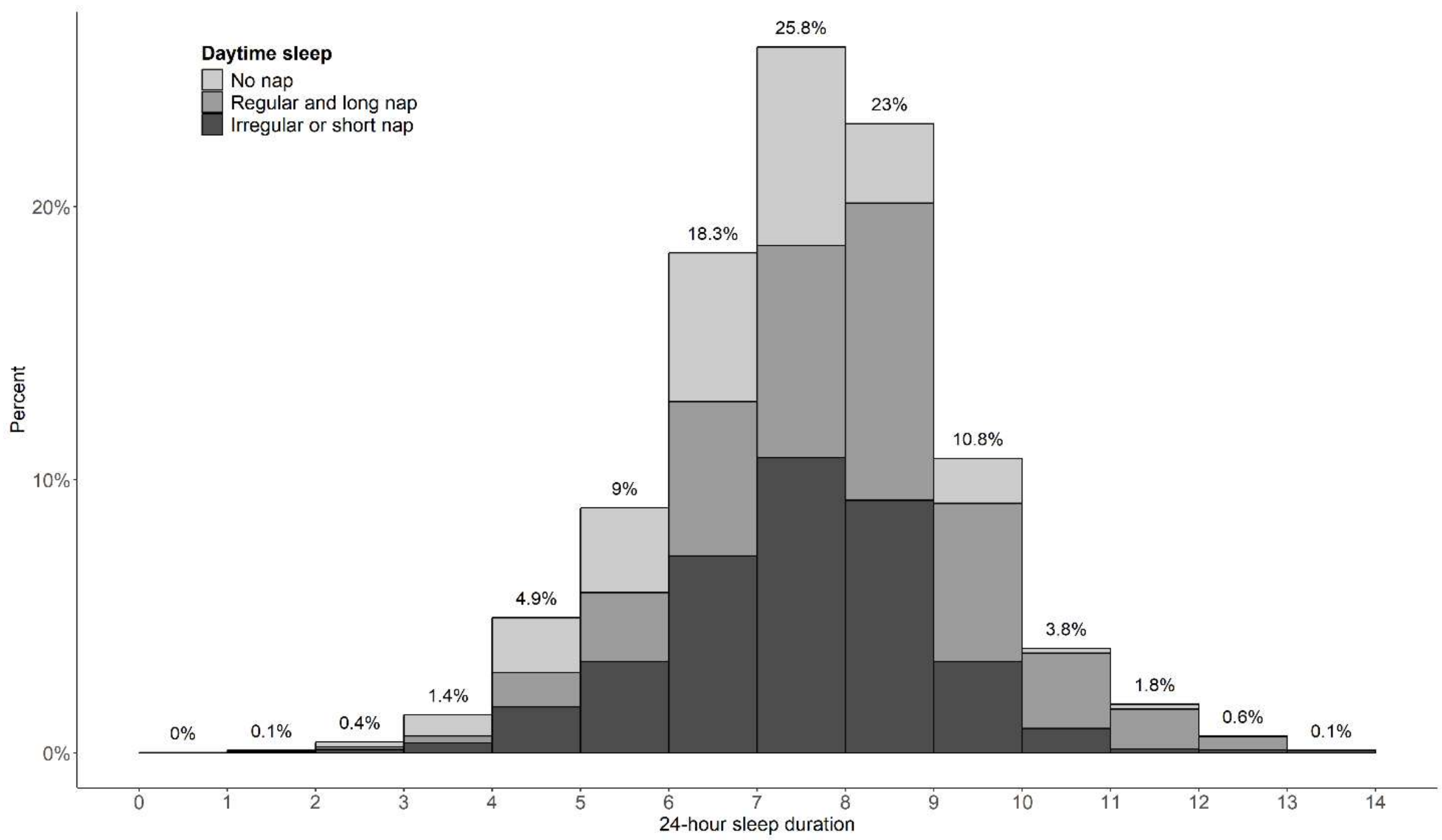

Figure S1- Distribution of 24-hour sleep duration including naps.
